# Pharmacological Modulation of Ubiquitin-Proteasome Pathways in Oncogenic Signaling

**DOI:** 10.3390/ijms222111971

**Published:** 2021-11-04

**Authors:** Anmol Sharma, Heena Khan, Thakur Gurjeet Singh, Amarjot Kaur Grewal, Agnieszka Najda, Małgorzata Kawecka-Radomska, Mohamed Kamel, Ahmed E. Altyar, Mohamed M. Abdel-Daim

**Affiliations:** 1 Chitkara College of Pharmacy, Chitkara University, Rajpura 140401, India; anmolsharma.860@gmail.com (A.S.); heenakhan0094@gmail.com (H.K.); amarjot.kaur@chitkara.edu.in (A.K.G.); 2 Department of Vegetable Crops and Medicinal Plants, University of Life Sciences in Lublin, 50A Doświadczalna Street, 20-280 Lublin, Poland; agnieszka.najda@up.lublin.pl (A.N.); malgorzata.kawecka@up.lublin.pl (M.K.-R.); 3 Department of Medicine and Infectious Diseases, Faculty of Veterinary Medicine, Cairo University, Giza 12211, Egypt; m_salah@cu.edu.eg; 4 Department of Pharmacy Practice, Faculty of Pharmacy, King Abdulaziz University, P.O. Box 80260, Jeddah 21589, Saudi Arabia; aealtyar@kau.edu.sa; 5 Department of Pharmaceutical Sciences, Pharmacy Program, Batterjee Medical College, P.O. Box 6231, Jeddah 21442, Saudi Arabia; 6 Pharmacology Department, Faculty of Veterinary Medicine, Suez Canal University, Ismailia 41522, Egypt

**Keywords:** cancer, ubiquitination, ubiquitin-proteasome system, deubiquitination, ubiquitin inhibitors

## Abstract

The ubiquitin-proteasome pathway (UPP) is involved in regulating several biological functions, including cell cycle control, apoptosis, DNA damage response, and apoptosis. It is widely known for its role in degrading abnormal protein substrates and maintaining physiological body functions via ubiquitinating enzymes (E1, E2, E3) and the proteasome. Therefore, aberrant expression in these enzymes results in an altered biological process, including transduction signaling for cell death and survival, resulting in cancer. In this review, an overview of profuse enzymes involved as a pro-oncogenic or progressive growth factor in tumors with their downstream signaling pathways has been discussed. A systematic literature review of PubMed, Medline, Bentham, Scopus, and EMBASE (Elsevier) databases was carried out to understand the nature of the extensive work done on modulation of ubiquitin-proteasome pathways in oncogenic signaling. Various in vitro, in vivo studies demonstrating the involvement of ubiquitin-proteasome systems in varied types of cancers and the downstream signaling pathways involved are also discussed in the current review. Several inhibitors of E1, E2, E3, deubiquitinase enzymes and proteasome have been applied for treating cancer. Some of these drugs have exhibited successful outcomes in in vivo studies on different cancer types, so clinical trials are going on for these inhibitors. This review mainly focuses on certain ubiquitin-proteasome enzymes involved in developing cancers and certain enzymes that can be targeted to treat cancer.

## 1. Introduction

**Figure 1:**
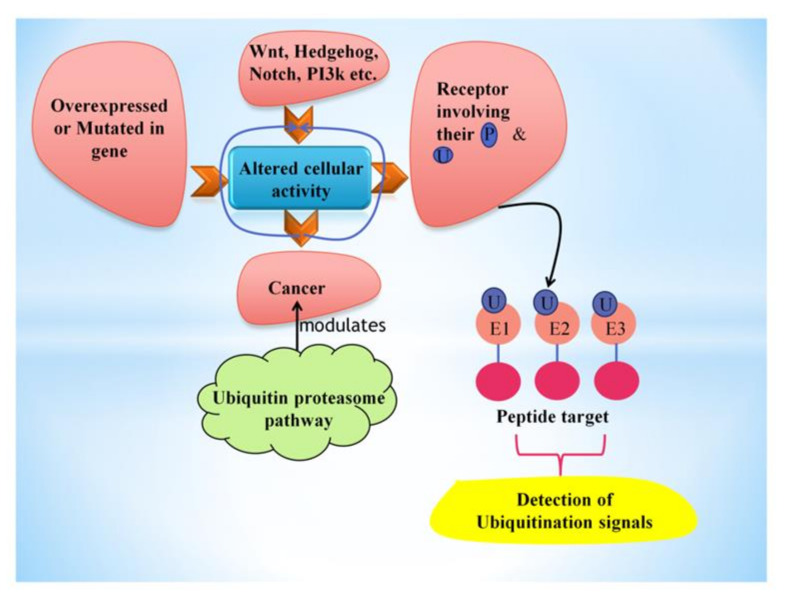
This figure shows the factors leading to the alteration of cellular activity, further causing cancer, which can be inhibited or influenced by the ubiquitin-proteasome pathway. Ubiquitin polymers attach covalently to peptide targets through a three-step (E1→E2→E3) conjugation cascade to detect particular ubiquitination signals.

Cancer is one of the dreadful diseases increasingly affecting the population worldwide. There is an aberrant gene function in cancer cells, which controls protein synthesis, cell growth, differentiation, and cell death. These activities are regulated by various pathways interconnected with each other to form a complex network. Alterations in these signaling pathways alter cellular activity’s progress that might lead to the over-production of proteins and uncontrolled cell growth, resulting in cancer [1]. One of the reasons for altered cellular activity is mutations in their genes and the overexpression of these mutated genes (e.g., gene amplification), which diverts the action from normal cellular activity. Downstream nuclear targets of cellular pathways, e.g., chromatin remodelers (EZH2), transcriptional factors (Myc and NF-κB), and cell cycle effectors (cyclins), are upregulated in cancer and also act as pro-oncogenic in tumor onset (Figure 1). The presence of tumor suppressors like p53, PTEN, p16, etc., in the body regulates cell death; therefore, the mutated suppressor genes ultimately lead to uncontrolled cell growth [2]. The regulatory processes that allow specific, rapid, and usually irreversible differences in cell sensitivity to ligands have evolved towards regulating receptor degradation and downregulation.

These mechanisms are often controlled by post-translational modifications of receptors involving their phosphorylation and ubiquitination [3]. In eukaryotes, protein degradation is essential for removing excessive proteins, e.g., enzymes and transcriptional factors (that are no longer required) or exogenous peptides transported in the cells. Two larger involved protein degradation systems present in cells are the autophagy-lysosome and the ubiquitin-proteasome systems [4]. The ubiquitin-proteasome pathway degrades nuclear and cytosolic proteins through an ATP- and ubiquitin-dependent process focused on the multicatalytic proteinase complex known as the 26S proteasome [5]. Ubiquitin polymers are formed, attached covalently to peptide targets through a three-step (E1→E2→E3) conjugation cascade, detecting particular ubiquitination signals. Targets can exist in the cytoplasm, nucleus, even on cell or nuclear membrane surfaces, or from the endoplasmic reticulum (ER) after their retrograde transfer back to the cytoplasm [6]. Overall, ubiquitination-proteasome-deubiquitination is an essential regulatory activity that keeps equilibrium in responses to the surrounding in vivo [6]. Different enzymes involved in the ubiquitin-proteasome pathway have distinct cell roles that regulate cell growth and death via triggering or degrading intermediates of another signaling pathway.

Notably, the ubiquitin-proteasome pathway is responsible for degrading tumor suppressor components and can influence cell differentiation in cancerous cells. In brief, the ubiquitin-proteasome pathway and its components have both benefits and detriments depending on their nature towards cellular pathways, which have been explained below in this review.

### Methodology

**Figure 2:**
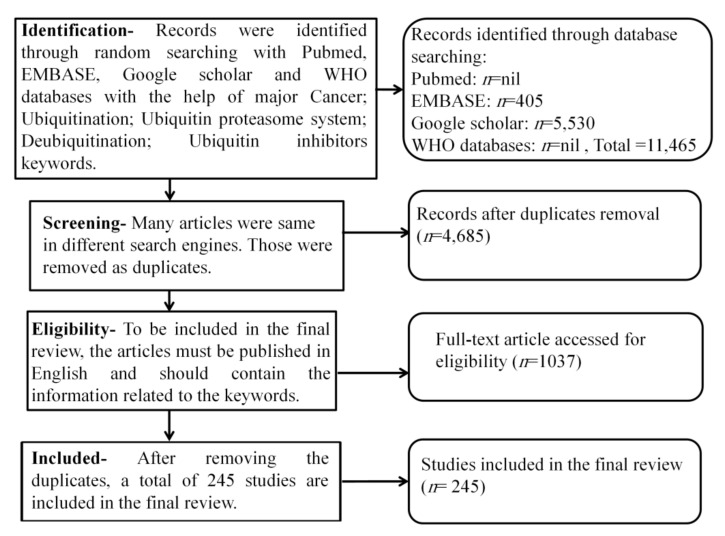
Flowchart of methodology.

A systematic literature review of PubMed, Medline, Bentham, Scopus, and EMBASE (Elsevier) databases was carried out with the help of the keywords like “cancer; ubiquitination; ubiquitin-proteasome system; deubiquitination; ubiquitin inhibitors” till March 2021. The review was conducted using the above keywords to collect the latest articles and understand the nature of the extensive work done on the modulation of ubiquitin-proteasome pathways in oncogenic signaling (Figure 2).

## 2. Molecular Biology of the Components in Ubiquitin-Proteasome System

### 2.1. Ubiquitin

Ubiquitin comprises 3.5 turns α-helix, 5-stranded β-sheet, and a small 310 helix [7]. In the human genome, there are four genes, namely, UBB, UBC, UBA52, and RPS27A, that encode ubiquitin. Genes UBB and UBC encode for the polyubiquitin molecules that give tandem repeats 3 and 9, respectively. However, genes UBA52 and RPS27A encode for a single copy of ubiquitin, which is fused with the ribosomal protein subunits via N-terminus, L40 S27a, respectively [8]. Ubiquitin is a 76 amino acid protein having seven Lys residues, and all of them can be ubiquitinated to form isopeptide-linked ubiquitin chains [9]. As data indicates, ubiquitin is modified by post-translational modifications; six out of seven Lysine residues (Lys6, Lys11, Lys29, Lys27, Lys33, Lys48, Lys63) of ubiquitin can be acetylated [10]. Phosphorylating sites are also present on the surface of ubiquitin-Ser 57, Ser20, Ser65, Thr7, Thr12, Thr14, Tyr59 [10,11]. Spotting of ubiquitin and ubiquitinated proteins are perceived by ubiquitin-binding domains, which form (autonomous) folding units within ubiquitin receptor proteins [12]. More than 20 ubiquitin-binding domain families can read the ubiquitin code within ubiquitin-binding proteins, and ubiquitin receptors that interact with other ubiquitin surfaces through non-covalent bonds [13]. Various protein ubiquitination activities are influenced by ubiquitin-binding domains, which include at least 16 known domains: UBA, UIM, MIU, CUE, DUIM, GAT, Ubc, UEV, NZF, A20 ZnF, UBP ZnF, UBZ, GLUE, UBM, Jab1/MPN and PFU (ubiquitin associated domain; ubiquitin interacting motif; motif interacting with ubiquitin; coupling of ubiquitin; double-sided ubiquitin-interacting motif; GGA and Tom1; ubiquitin C; ubiquitin E2 variant; npl-4 Zinc finger; A20 zinc finger; ubiquitin-binding domain zinc-finger; ubiquitin-binding zinc finger; GRAM-like ubiquitin-binding in EAP45; ubiquitin-binding motif; and PLAA family ubiquitin, respectively) [14]. The ubiquitin-binding domain attachments have an essential influence on therapeutic strategy. For example, in a study, displacement of Ub from the Zn-finger ubiquitin-binding domains of HDAC6 (main cytoplasmic deacetylase in mammals) can be a useful target for multiple myeloma or other disease therapy [15].

### 2.2. Proteasome

**Figure 3:**
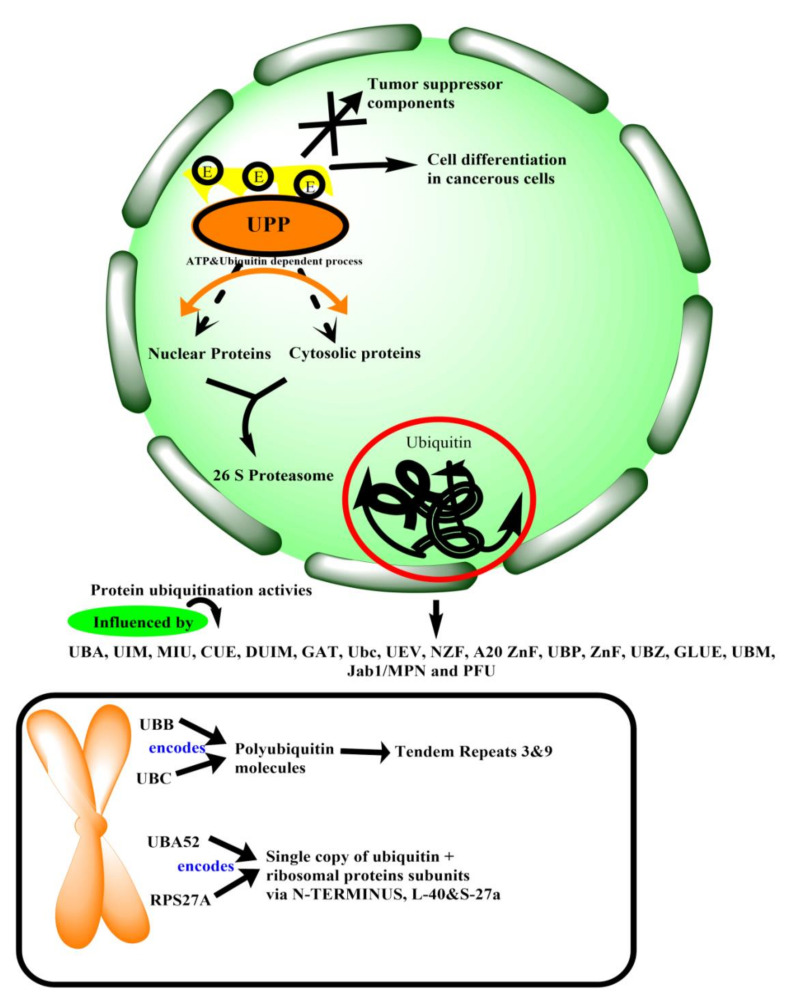
Illustration of ubiquitin in cancerous cells and human genome encodings, i.e., UBB and UBC encode for polyubiquitin molecules and UBA52 and RPS27A encodes for single copy of ubiquitin and ribosomal proteins subunits. Ubiquitin-proteasome pathway degrades nuclear and cytosolic proteins through an ATP- and ubiquitin-dependent process. Ubiquitin polymers are formed by covalent attachment of E1, E2, E3 which involves different enzymes having distinct cell roles that regulate cell growth and death via triggering or degrading signaling pathways. The UPP is responsible for degrading tumor suppressor components and can influence cell differentiation in cancerous cells.

Proteasome (also known as 26S proteasome) is a protein degradation machine in eukaryotic organisms. It is a 2.5 MDa complex that comprises approx. 33 different subunits arranged into two subcomplexes: a barrel-shaped proteolytic core particle (alias the 20S proteasome; CP) and one or two terminal(s) 19S regulatory particle(s) (alias PA700; RP) [16,17,18]. This holoenzyme’s proteolytic active sites are present within the core of the 20S core particle, consisting of four heptameric rings [18]. It forms a narrow axial pore, which does not allow folded protein or even unfolded large polypeptides to pass through, thus protecting normal body proteins from degrading (Figure 3). The regulatory particle(s) controls the opening of these pores [19,20], covering one or both ends of 20S core peptidase and transferring client protein into the degradation chamber [21]. The 19S RP seems to recognize ubiquitylated substrate proteins and is considered to serve a role in protein unfolding and translocating it into the interior of 20S CP. Catalytic threonine residues are present over the core particle’s surface, composed of two β-rings [22]. There are two subunits of the 20S- α-type [α1, α2, α3, α4, α5, α6, α7, α8] and β-type [β1, β2, β3, β4, β5, β6, β7, β1i, β2i, β5i, β5t] [18]. The 20S CP/20S proteasome (of 730kDa) is a well-arranged protein complex formed by four stacked hetero-heptameric rings, which comprises of 7 α-type subunits or seven β-type subunits in a symmetric configuration of α1−7/β1−7/β1−7/α1−7 C2 [23]. The 19S regulatory particle(s) of ~930 kDa is formed by at least 19 integral subunits of molecular masses ~10 to 110 kDa. It can be separated into two subcomplexes: the base and the lid [24]. The base is made with a ring of AAA-ATPases (Rpt1-6) with four non-ATPase subunits (Rpn1, 2, 10, 13) and contact with CP. The peripheral lid consists of Sem1 (alias Rpn15/Dss1) and an additional 10 ATPase subunits with different functions [25]. The contact between the base and lid is balanced by the subunit Rpn10 [22]. The scaffolding subunits (except for Rpn15) involves protein–protein interacting motifs called PCI [proteasome-CSN (COP9 signalosome)-eIF3 (eukaryotic translation initiation factor 3)] domains [26,27].

### 2.3. Deubiquitinase

DUBs are an enzyme that can reverse the activity of ubiquitination or ubiquitin-like modifications of substrate proteins, thereby protecting the protein from degrading [28,29]. DUB antagonizes protein ubiquitination similarly to phosphatases’ role in the kinase/phosphatase regulating pathway(s) [30]. Humans’ genomic system encodes for nearly 100 DUBs that are ubiquitin-specific and divided into five structurally unique DUB families. DUBs are categorized into five classes, including the ubiquitin-specific protease (USP) with 54 members, the ovarian-tumor proteases with 16 members, the ubiquitin C-terminal hydrolases with four members; the Josephin family with four members [31]. The fifth DUB family is MIU containing a novel DUB family (MINDY) with four members [32]. A Zn-dependent JAB1/MPN/MOV34 metalloprotease DUB family also exists with 16 members [33]. The ubiquitin removal step appears to be a highly regulated sequence of action connected with uncountable cellular functions. DUBs are implicated in maintaining cell cycle stages, double-stranded sliced repair, and the M/G 2 checkpoints, averting protein degradation and transcriptional activities. It is also involved in apoptosis, microbial pathogens, viral precursor protein, kinase activation [34].

### 2.4. Ubiquitination

Ubiquitination can be defined as a cascade of events by three enzymes performing their respective action to attach selected substrate proteins with ubiquitin for prior modifications. It is one of the essential protein modifications involved in cellular signaling and homeostasis control. The process initiates with enzyme E1 for activating ubiquitin. The activated ubiquitin is then transferred to the E2 enzyme, and ubiquitin linkage forms (mono-ubiquitination, polyubiquitination, branched ubiquitination). Detachment of ubiquitin from E2 can trigger attachment with substrate protein and directly approach for proteasome degradation [35]. Otherwise, the E2 enzyme serves to identify specific E3 ligases where it transfers the substrate protein. Then, ligase releases substrate protein to proteasome or are obstructed by deubiquitinase, leading to protein survival. The whole process can be called as ubiquitination of substrate protein. All the enzymes are explained in detail below:

#### 2.4.1. E1 (Ubiquitin-Activating Enzyme)

Gathered literature suggests there are only two E1 enzymes in humans, i.e., a non-specific Uba 1 enzyme, which can activate ubiquitin for all ubiquitin-dependent reactions, and an organ-specific enzyme UBE1L2 [36,37,38]. Uba1-E1 comprises of four building blocks, in which first is an adenylation domain labeled ‘AAD’ (404–594) (for ATP-Ub binding) and ‘IAD’ (1–169) for active and inactive adenylation, respectively. Second is the catalytic cysteine half domain-containing E1 active site Cyt [CC (169–268) and CCD (594–860)] incorporated within each adenylation domain. The third block contains 4HB (helix bundle) (268–356) depicting the second insertion in IAD, and the fourth includes the C-terminal ubiquitin-fold domain, UFD (926–1024), which selects specific E2s for ubiquitin [39,40]. Recently found UBE1L2, i.e., ubiquitin activating enzyme E1-like protein 2, share its 40% identity with Ube1. There are two essential conserved domains of UBE1L2: the highly protective ATP-binding domain (amino acid 467–474; GXGXXGCE) and the putative active site domain (amino acid 623–631; PXCTXXXP) encompassing Cys-625, which can form thioester links with ubiquitin [40,41].

#### 2.4.2. E2 (Ubiquitin-Conjugating Enzyme)

Humans have approximately 40 E2s that support the transfer of ubiquitin or ubiquitin-like proteins (e.g., NEDD8 and SUMO), and most of them are double the size of ubiquitin. The E2 enzymes are involved in tagged protein regulations for their localization, degradation, and other functions [42]. Some E2s have only a catalytic domain named ‘Class 1′ whereas some have either N- ‘Class 2′ or C- ‘Class 3′ terminal extensions or contain both ‘Class 4′ [42]. The E2 enzymes are “Ube2A, Ube2B, Ube2C, Ube2D1, Ube2D2, Ube2D3, Ube2D4, Ube2E1, Ube2E2, Ube2E3, Ube2G1, Ube2G2, Ube2H, Ube2J1, Ube2J2, Ube2K, Ube2L3, Ube2N, Ube2NL, Ube2O, Ube2Q1, Ube2Q2, Ube2QL, Ube2R1, UbE2R2, Ube2S, Ube2T, Ube2U, Ube2V1 Ube2V2, Ube2W, BIRC6” [43].

#### 2.4.3. E3 (Ubiquitin-Ligase Enzyme)

The ubiquitin attached with E2 (linear or chain) is transferred to ubiquitin ligase E3, and the detached ubiquitin binds with substrate protein and ligase enzyme. According to the mechanism followed for the transfer from an E2 enzyme to substrate, E3s are classified into:

(i) Really interesting new gene [RING] finger domain-containing ligases (BIRC7, Brca1, Cb1-b, cIAP1, IDOL, mdm2, SIAH1, RAD18, RNF4, TRAF6): The RING E3 enzymes are differentiated due to their RING or Ubox (CHIP) fold catalytic-domain which encourages the direct linking of ubiquitin from E2 with the substrate [44,45,46]. There are more than 600 RING finger E3s encoded in the mammalian genome. Structurally, it is a zinc coordinating domain that consists of spaced cysteine and histidine residues which promote E2 dependent ubiquitylation [47].
(ii) Homologous to E6-associated protein C terminus [HECT] domain-containing ligases (SMURF1, NEDD4.1, HUWE1, E6AP): It consists of 28 members in human and based on similarities in the N- terminus domains, 15 members out of 28 can be divided into two subfamilies. The most prominent and well-studied category is the NEDD4 subfamily consisting of nine members, which are characterized by the presence of C2 and WW domains. Another subfamily is the HERC E3 ligase enzyme consisting of six members and shares one commonality, i.e., one or more RCC-like domains (regulators of chromatin condensation 1-like domains) [48].
(iii) RING between RING (RBR) domain-containing ligases: Structurally, the RBR module has three Zn^2+^ binding motifs, a RING1 domain which interacts with E2 then followed by IBR, which is in-between RING1 finger domain and the RING2 domain of which catalytic cysteine is involved. There are 14 RBRs in humans; among these, only three members that are well understood are Parkin, HHAR1, and HOIP [49,50].

## 3. Physiological and Pathological Role of UPS in Human Body

**Figure 4:**
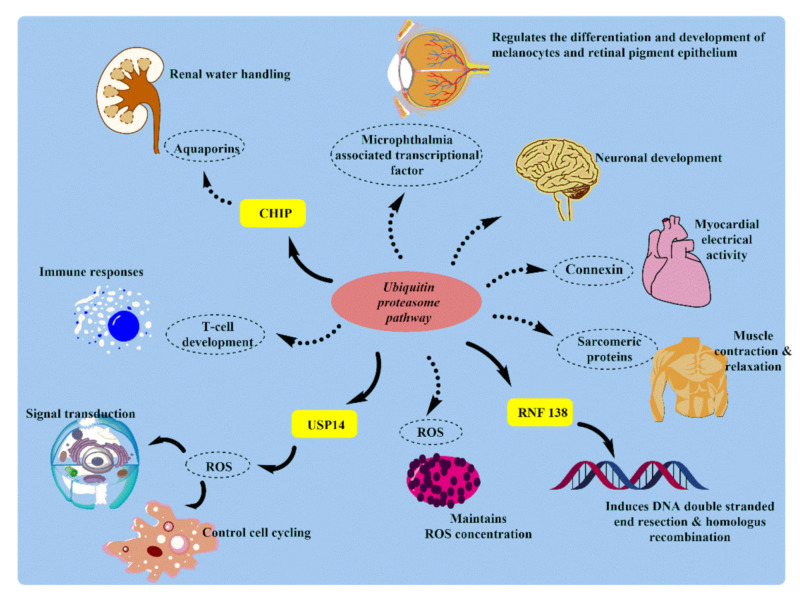
This figure summarizes the pathophysiological role of UPP in various diseases and indicates the dysregulated enzyme in the respective condition.

The ubiquitin-proteasome system plays a crucial role in protein quality control, cell cycle control, and signal transduction. Proteasome in the cytoplasm is mainly concentrated near the centrosome, which indicates its activity at the cellular level. Ubiquitin-proteasome pathway (UPP) components are seen in very specialized cells, including neurons of both pre-and post-synaptic knobs. In every physiological process, the ubiquitin-proteasome pathway exists, and system variation can lead to the onset or progression of human disease. These diseases include cancer, metabolic syndrome, neurodegenerative diseases, inflammatory disorders [51], infection [52], and muscle dystrophy [53] (Figure 4).

In the nervous system, the ubiquitin-proteasome system exemplified its importance in regulating many aspects of synaptic activity such as spinogenesis, axon growth, pre-synaptic neurotransmission, synaptic scaling, long-term potentiation, apical dendrite outgrowth/polarization, synapse formation, dendritic arborization, and elimination [52,54,55].

In the cardiovascular system, like any other proteins within cells, cardiac myofibrillar proteins are also constantly being broken down and rebuilt. By investigating proteolytic systems’ role in skeletal muscle wasting and atrophy via the ubiquitin-proteasome system, it was found that the ubiquitin-proteasome system is essential for the degradation of the sarcomeric proteins [56,57,58]. In the muscular system, muscle atrophy/wasting is the main reason for the increase in protein degradation by autophagy, including the ubiquitin-proteasome system. During investigating the role of proteolytic systems in skeletal muscle wasting [59] and atrophy, it was found that not many enzymes are known yet. In several muscle wasting and atrophy cases, the ubiquitination system is activated and upregulated, increasing proteasome activity. For example, the highly expressed muscle-specific ubiquitin ligases (MuRF-1 and MAFbx/atrogin-1) recently came into view, which paves the way for the process of atrophy.

In spermatogenesis, protein’s selective degradation is reported in various data related to reproductive processes, especially spermatogenesis, fertilization, and testosterone biosynthesis [60]. Spermatogenesis can be represented as a complex succession of cell division and differentiation events resulting in spermatozoa’s continuous formation. In the kidney, microarray analysis showed that passive Heymann nephritis is associated with increased expression of genes which encodes for ubiquitin-conjugating enzymes and deubiquitinase enzymes. This analysis supports the UPS role in kidney functioning and disorders [61].

The ubiquitin-proteasome system’s importance extends from the kidney to the nephrons’ outline in glomerular cell identity and function, glucose reabsorption, erythropoiesis, and salt-water balance. The cortical collecting duct’s principal cells express various aquaporins on its surface, maintaining body water balance [62]. However, genomic stability maintenance in situations like DNA damage critically relies on quick recognition and healthy repair of damage [63,64].

## 4. Role of Ubiquitin-Proteasome Pathway in Cancer

Cancer is one of the leading causes of death globally for ages. There have been many types of research on cancer for its treatment and finding new targets, but no successful results have been found. Mortality data collected since 2016 by The National Center for Health Statistics and an estimate for 2019 indicates that approximately 626,000 cancer deaths and over 1,762,000 new cancer cases occur in the US [65]. Cancer can be defined as high cell proliferation and low cell death due to disturbance in the cell cycle, a mutation in existing cells, activation of tumor promoter genes, inactivation of the tumor suppressor, irregularities in a feedback mechanism, deregulation in cell cycle pathways, etc. [66]. The tumor suppressors are somewhat targeted by UPP-related enzymes and inhibit their role in cell death. Various studies mentioned below show the ubiquitin-proteasome pathway’s involvement via the above mediators/pathways, leading to cancer one way or another.

### 4.1. Colorectal Cancer

**Figure 5:**
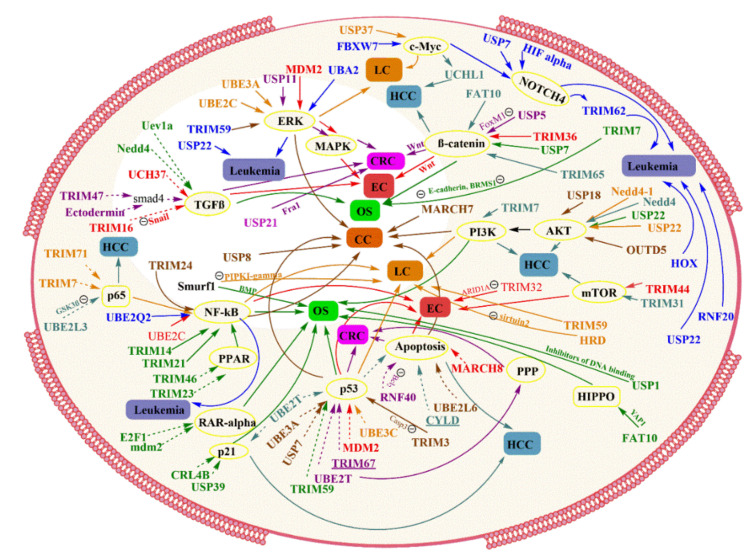
The figure represents various physiological roles of UPP and the enzymes/proteins involved. CRC; colorectal cancer, EC; esophageal cancer OS; osteosarcoma, LC; lung cancer, HCC; hepatocarcinoma cells, CC; cervical cancer, leukemia.

Colorectal cancer (CRC) is the third most prevalent cause of death from cancer in the US [65]. Lynch syndrome, serrated polyposis syndrome, MUTYH-associated polyposis, familial adenomatous polyposis, etc., are the commonly found hereditary colorectal cancer, and polyposis syndromes. Colorectal cancers can be associated with genetic inheritance, African-American ethnicity, inflammatory bowel disease, red meat/processed meat, abdominal radiation, cholecystectomy, also includes unhealthy lifestyle, etc. [67]. Mutations and dysregulation in the normal signaling pathways cause cancer. Pathway TGF-β involves a large family of proliferation and differentiation factors which include activin and inhibition. Transmission of signals occurs via Types I and II serine/threonine kinase receptors [68]. Ligand binding initiates and triggers the activation of Type I kinase by the Type II receptor kinase. Then, the signal is passed from the Type I receptor to an intracellular mediator of TGF-β, Smads, which initiates Smad signaling for nuclear translocation and activation of specific gene expression. Nearly 13% of colorectal carcinomas are caused due to replication error (or microsatellite instability) phenotype on inactivation and restoration of receptor Type II, resulting in low tumorigenicity. Among Smads, Smad 6 and 7 act as an inhibitor of Smad, which blocks TGF-β signaling by competitively associating with Type I or directing it towards Ub-mediated degradation [69], increasing cellular growth (Table 1). Many ligases like Smurf1, Smurf2, Nedd4-2, arkadia, etc., are involved in TGF-β signaling, but E3 ligase ectodermin is an enzyme whose altered expression can lead to tumor formation via inhibiting Smad4, thereby blocking the TGF-β pathway [70]. Aberrant activation of pathway Wnt/β-catenin in the colorectal region can cause uncontrolled tumor growth, invasion, and angiogenesis (Table 1). In normal human cell regulation, a ligase enzyme FBXW7 prevents this oncogenic pathway from causing this problem. FBXW7 ubiquitinates a novel component of Wnt-signaling, FoxM1, after phosphorylated by glycogen synthase kinase 3. But in cancer, the situation is reversed by enzyme USP5 that deubiquitinate and prevents the degradation of FoxM1 [71,72]. USP11 offers a relevant role in cancer progression through multiple pathways. This enzyme proliferated and promoted metastasis of colorectal cancer in vitro and in vivo through PPP1CA-ERK/MAPK pathway [73]. An activator protein-1 (AP-1), precisely its member Fos-related-antigen-1 (Fras 1), has shown a tumorigenic role in many cancers. Usually, the AP-1 is mediated through the ERK pathway because Ras-ERK signals to constrain the proteasomal degradation of member the Fra-1. The Ras-ERK function can be achieved through an enzyme called deubiquitinase which can change the fate of protein from degrading, and in this case, UPS21 protects Fra-1, which promotes cellular growth in colorectal cancer [74] (Figure 5).

The ubiquitin-proteasome system participates in the DNA damage and repair pathway through UBE2T, a member of the ubiquitin-conjugating enzyme. In colorectal cancer, UBE2T role is defined as a promotor of tumor progression and metastasis. The exact pathway is still to be found because more than one pathway, “p53 pathway”, “pentose phosphate pathway,” etc., was noticed to be involved in cell line culture. Generally, cell homeostasis is maintained by p53 expression, and p53 activation can activate or deactivate other genes that regulate cell cycle arrest, apoptosis, metastasis, angiogenesis, and DNA repair [76]. As p53 degradation occurs through ligase mdm2, therefore the presence of ligase mdm2, which will increase cell growth and decrease cell apoptosis, eventually give rise to colorectal cancer malignancies. A ubiquitin ligase, tripartite motif-containing protein (TRIM) 67, links with the C-terminal of p53 and inhibits p53 degradation by mdm2; therefore, TRIM 67 is silenced in colorectal cancer. TRIM 47 is also seen to increase the ubiquitination and degradation of Smad4, blocking its inhibitory action on colorectal cancer. This activity occurs through C-C motif chemokine ligand 15 and C-C motif chemokine receptor 1 (CCL15-CCR1), promoting tumor growth and cell progression [75]. H2B ubiquitin ligase RING finger protein 40 showed to be a tumor promoter in various human colorectal cancer cell lines. Knocking down of RING finger protein 40 triggered apoptosis in cells through downregulating anti-apoptotic factor Bcl-2. Ligase enzyme RING finger protein 40 actively mediates the monoubiquitination of H2B and may exert pro-tumorigenic function in cell activity [140].

### 4.2. Esophageal Cancer

Esophageal cancer is the eighth most common cancer worldwide and the 6th leading cause of cancer-associated death in 2012. Histologically, there are two subtypes: esophageal squamous cell carcinoma (ESCC) and esophageal adenocarcinoma [141,142]. Mutated genes responsible for esophageal squamous cell carcinoma are MLL2, NFE2L2, NOTCH1, TGFBR2, and ZNF750, whereas esophageal adenocarcinoma is ARID1A, CDKN2A, ERBB2, SMAD4, and TP53 [143]. UBE2C (conjugating enzyme) promotes the ubiquitination of mitotic checkpoint genes; therefore, its overexpression emphasizes removing the inhibitory signal of mitotic spindle checkpoint in cells [77]. UBE2C normally functions as pro-apoptotic and anti-proliferative, but its expression is decreased by ECRG4 (esophageal cancer-related gene 4) in esophageal squamous cell carcinoma via NF-κB signaling. However, the mechanism in detail is yet to be explored [144].

Main overexpressed E3 enzymes found in esophageal squamous cell carcinoma are mdm2 (RING-type E3), TRIMs (RING-type E3), CUL3 (cullin subunit of the CRL3 E3), SKP2 (F-box protein, substrate receptor of CRL1 E3 (SCF complex)), CDC20 (substrate adaptor of the APC/C E3 complex (early mitosis)), KEAP1 (substrate receptor of the CRL3 E3) [145]. TRIMs are a specific type of E3 ligases characterized by their domain structure RING finger, and their activity seems to be regulated by β-catenin. However, TRIM36 is recently found to have relevance in esophageal cancer through stabilization of β-catenin, where the signal for β-catenin accumulation is transmitted via the Wnt signaling pathway [78]. Another TRIM categorized ligase that gave oncogene effect in esophageal cancer is TRIM44. Research shows it exhibit its role in esophaga-gastric cancer, where the mechanistic function responsible was suspected to be mTOR. Although the responsible pathway for the tumor progression and metastasis of overexpressed TRIM44 has not been identified, mTOR can be explored to start with [79].

Similarly, the proliferation, invasion, and migration are promoted by TRIM16 via TGF β and Snail pathway, which regulates epithelial-mesenchymal transition in esophageal cancer cell lines [80]. Smurf2 acts as a ubiquitin ligase for Smad, and its expression is higher in tumor cells, mainly at the tumor front, where the proliferation in esophageal squamous cell carcinoma is higher [146]. Most of the Wnt signaling factors involved in the post-translational modification are modulated through ubiquitination and deubiquitination.

Usually, the ubiquitination of target proteins leads to proteasomal degradation of Wnt-signalling factors like β-catenin, glycogen synthase kinase 3, Axin, and Dvl. Fz-7 is one of the Wnt receptors seen to be upregulated in esophageal squamous cell carcinoma patients. Its overexpression induces the activity of β-catenin, mesenchymal markers, and epithelial markers [147]. Binding of Wnt/Fzd activates dishevelled (Dvl) that avert the phosphorylation and ubiquitination of β-catenin. This leads to β-catenin stabilization and accumulation in the cytoplasm, further activating transcriptional activity for cellular growth [148]. In cell lines, analysis of RNF113A showed its overexpressed feature in esophageal squamous carcinoma cells, but its mechanistic approach is not yet found. Research has not been done on RNF113A, but previous studies suggest the involvement of epithelial-mesenchymal transition property in the process [81]. A ubiquitin ligase (MARCH 8) has been observed to be overexpressed in preneoplastic and neoplastic esophageal tissues. The MARCH 8 silencing activated the cell death process in the cell cycle by increasing sub-Gο and G2/M presence and lowering the S-phase population, which induces apoptosis [82]. Mdm2 act as ligase for p53, which inhibits its activity and result in tumor growth. There are other factors as well which are responsible for affecting p53 activity even in the absence or presence of mdm2. An ankyrin repeat-containing protein, gankyrin, associates with the subunit of ATPase of 26S proteasome, increasing the association between ubiquitinated p53 and mdm2 with the proteasome [83]. A deubiquitinating enzyme “ubiquitin carboxyl-terminal hydrolase 37” is involved in the activated TGF-β receptor type-I’s deubiquitination. Thereby preventing it from degradation via the proteasome, which results in TGF-β dependent gene over-expression. Hence ubiquitin carboxyl-terminal hydrolase 37 plays an oncogenic factor in esophageal cancer [84].

### 4.3. Osteosarcoma

Osteosarcoma is an autosomal dominant form of bone cancer with intrinsic osteoid production. Though the exact cause of osteosarcoma is unknown, the disease can be multifactorial with genetic and environmental factors [149]. The inactivation of the tumor suppressor gene p53, which regulates cell cycle progression in the presence of DNA damage, may result in osteosarcoma. Other genes involved in the p53 pathway like mdm2, p14ART, and CDK4 may develop osteosarcoma in a person [150]. The ubiquitin-proteasome system mainly regulates proteins that manage bone cells, osteoclast, and osteoblast (responsible for aged bone resorption and new bone formation, respectively) [151]. The critical factors controlling osteoblast differentiation from mesenchymal stem cells are bone morphogenetic protein-2, Wnt/β-catenin pathway, and transcriptional factors- Runx2, activating transcription factor 4, and JunB. The ubiquitin ligases substrates that affect bone metabolism are Smurf1 (Smad1, BMP-2, Runx2, Jun B, Traf6), β-TrCP (β-catenin, Smad4, ATF4, CYLD, NRF2, GHR), Fbx112 (p57), Chip (Smad1), Keap-1-Cul3-Rbx1 (Bcl-2, IKK, Nrf2), c-Cbl (EGFR/FGFR, α5 Integrin, Lyn/Fyn) [152,153].

The ubiquitin-conjugating enzyme E2 variants (Uevs) are seen to be involved in the bone morphogenetic protein signaling pathway, and its inactivation will negatively affect the functioning of bone cells. Uev1A is a novel OS regulator linked with (E2-E3 complex) UbcH5B-Smurf1 and further enables the ubiquitination and degradation of osteosarcoma promoting factor Smad1. Uev1A has also shown its serious role in preventing osteosarcoma by promoting osteoblast differentiation; therefore, inactivation of Uev1A might result in osteosarcoma [85]. Nedd4 is an essential modulator of p-Smad1 (phosphorylated-Smad1) in both bone morphogenetic protein-1 and TGFβ1 (Table 1). Nedd4 overexpression suppressed the bone morphogenetic protein-induced trans-differentiation process in cells and promoted TGFβ1 induced p-Smad1 degradation by polyubiquitination. Also, the suppressed expression of nedd4 speeds up the osteoblast differentiation process in osteosarcoma cells [86]. In cancer, the Wnt/β-catenin signaling pathway can promote epithelial-mesenchymal transition in cells via the canonical Wnt pathway responsible for β-catenin accumulation in the cytoplasm. Besides, β-catenin is translocated into the nucleus, where different interaction of β-catenin regulates the downstream gene expression. β-catenin also recruits transcriptional factor, Snail, that functions as a key promoter in epithelial-mesenchymal transition, downregulates epithelial gene, and upregulates mesenchymal genes. USP7 acts as a DUB for β-catenin and prevents its degradation by ubiquitin-proteasome in osteosarcoma cells [87,154,155].

The basic “helix-loop-helix” transcriptional factors inhibit differentiation, thereby sustaining stem cell fate, and are opposed by the ‘inhibitors of DNA binding.’ The ubiquitination and degradation of DNA binding inhibitors occur in differentiated tissues but appear to escape degradation in many neoplasms. A DUB enzyme, USP1, and the stability of stem cell-like property in osteosarcoma promote the protein stability of “inhibitors of DNA binding” by binding & deubiquitinating it [88]. In cancers, the function of NOTCH signaling may vary according to cell context as it can act as both a tumor suppressor and oncogenic. It works by reciprocal inhibition of two NOTCH downstream effectors: Deltex1 (a RING finger E3 ligase) and a NOTCH’s primary target, HES1 (a helix-loop-helix repressor family). Deltex1 shows an inhibitory effect on the NOTCH/HES1 pathway through binding with NOTCH intracellular domain, leading to ubiquitination and degradation of NOTCH receptors.

On the contrary, HES1 directly binds with the promotor of Deltex1 and causes transcriptional inhibition of Deltex1 [89]. HES1 promotes invasiveness and metastasis in vivo; therefore, an over-expression of HES1 and under-expression of Deltex1 are seen in osteosarcoma conditions. The overexpression of a highly regulated pathway like Hippo/YAP1 is closely related to tumorigenesis, regulated through the ubiquitin-proteasome pathway.

Ubiquitin-like protein, FAT10, initially acts as deubiquitin and protects the degradation of component YAP1 in the pathway, promoting aggressive growth of tumors in osteosarcomas [90]. The discovery of mdm2 acting like ligases has revealed regulations of many oncogene proteins in tumors. Its involvement has been seen in retinoic acid receptor alpha (RARα), which mediates all-trans retinoic acid biological effects (ATRA). Mdm2 leads to the degradation of RARα, thereby impairs ATRA-induced osteogenic differentiation in osteosarcoma cells [156]. USP39 is known to be a critical eukaryotic gene expression and classified under deubiquitinase. In research, USP39 knockdown has positively influenced cell apoptosis by arresting cell division at the G2/M phase via the p21 pathway. Due to its involvement in maintaining spindle checkpoints and supporting cytokinesis, its overexpression led to cells’ continuous growth [91]. Ligase TRIMs correlations are seen in osteosarcoma and TRIM46, TRIM21, TRIM14, and TRIM23 following the NF-κB pathway [92]. Other components concerned with osteosarcoma are reducing p53 and E-Cadherin, which are followed by TRIM59 and TRIM7 [93,94]. One of the important promoters of tumor growth in osteosarcoma is the PI3K/AKT pathway, and a lot of enzymes are known, involved in various cancer forms. USP22 and UBE2T are related to PI3K/AKT pathway and promote cell progression and invasiveness in osteosarcoma [95,96].

### 4.4. Lung Cancer

Lung cancer is the second most common cancer, with an estimated 1.6 million mortality rate each year. The cause of lung cancer may differ across the world, reflecting the geographical differences in air quality and tobacco use [157]. Most of the patients (approx. 85%) have histological subtype NSCLC (non-small cell lung cancer cells), among which lung squamous cell carcinoma (LUSC) and lung adenocarcinoma (LUAD) are the most common [158]. Zhao, X.C. et al., studied the ubiquitylation pathway’s involvement in modulating non-small cell lung cancer cell growth and found cell division cycle 34 (ubiquitin conjugating enzyme) essential candidates for cell. Cell division cycle 34 is increased in nearly 66% of non-small cell lung cancer cells in smoker patient tumor tissues than non-smoker patients [159]. TGF-β (which serves as a negative growth regulator), when modulated by inhibitory-Smads (Smad6 and Smad7), interrupts the receptor-mediated phosphorylation of Smad2/3 and represses the signaling pathway. Smad6 is seen to be overexpressed in tumor cells of lung cancers [160]. Smad ubiquitination regulatory factor 1 (Smurf1) is an E3 ubiquitin ligase that contains the WW domain, C2 domain, and HECT domain. In the study, it was observed that the Smurf1 positive non-small cell lung cancer patients have better chances at survival; therefore, the negative regulation of Smurf1 results in lung carcinogenic due to interruption in TGF-β signaling [161].

A PTEN gene, tumor suppressor, encodes a lipid phosphatase that dephosphorylates the secondary messenger phosphatidylinositol 3,4,5-triphosphate and opposes the action of PI3K (phosphatidylinositol 3-kinase). Hence, the increase and activating mutations in the PI3K gene or loss of PTEN gene leading to PI3K activation have been observed in non-small cell lung cancer cells. In lung cancer, NEDD4-1 as E3 ligase handles PTEN stability and gives a mechanism that contributes to the inactivation of the PTEN gene [97]. NEDD4 has also proved its oncogenic activity via EGFR in lung cancer cell lines [98]. The p16INK4a is a tumor suppressor, in which one protein is encoded by one of its locus INK4/ARF and is absent in various cancer, including non-small cell lung cancer cells. One of the mechanisms which silence the INK4/ARF is E3 ubiquitin ligase and transcriptional cofactor E6AP (UBE3A). E6AP induces the expression at the transcriptional level by restraining CDC6 transcription, a gene that encodes an essential repressor of the INK4/ARF locus [99]. E3 ligase HMG-CoA reductase degradation protein 1 (HRD1) deficiency induces Sirtuin2 upregulation. Sirtuin protein is responsible for the regulation of mitotic cell exits and, therefore, cell cycle maintenance. In lung cancer, the sirtuin2 expression is downregulated due to a negative correlation with HRD1 expression [100]. A deubiquitinating enzyme stabilizes the oncoprotein c-Myc (DUB) USP37 through direct binding and ceases its degradation. Clinically, USP 37 regulates cell proliferation and is upregulated in human lung cancer tissues, which are positively correlated with c-Myc protein expression [101]. Various evidence states that several TRIM (E3 ligase) act as regulators of NF-κB and are involved in ubiquitinating proteins at different NF-κB pathway steps. Usually, the canonical activation of NF-κB depends on IκB degradation, but some cases suggest that p65 protein degradation is required for the termination of NF-κB transduction; therefore, p65 may as well play a role in NF-κB signaling.

In non-small cell lung cancer cells, TRIM7 interacts with p65, thereby promoting its ubiquitin-mediated degradation, and therefore TRIM7 regulates NF-κB signaling [102]. Another TRIM acting like ligase which promotes proliferation through NF-κB is TRIM71. It is highly expressed in the non-small cell lung cancer cell line due to its involvement in reducing IκB [103]. In non-small cell lung cancer cells, an oncogenic role of the UBE2C [162] conjugating enzyme is observed. UBE2C can regulate gene expressions associated with apoptosis, angiogenesis, and tumor growth via the ERK pathway, according to array analysis. This enzyme was examined in a culture, and researchers discovered that it helps trigger apoptosis in cells by directly regulating phospho-ERK1/2 [104]. The conjugating enzyme has a specific role in p53 activation for oncogene triggering. It appears that overexpression of the enzyme UBE3C reduces the presence and function of AHNAK. Mechanistically, UBE3C interacts with AHNAK and disturbs its complex with p53, which blocks its inhibitory action on tumor growth, resulting in enhanced stemness [105] (Table 1). USP22 has been implicated in early-stage non-small cell lung cancer, but its mechanistic approach has not been identified yet. However, previous studies on other cancers suggest its role in the AKT pathway [107]. Many TRIMs have shown their oncogenic function in lung cancer involving TRIM 59. In the cell line culture of lung cancer, the absence of TRIM59 triggered cell cycle arrest at the G2 phase, which shows its tendency to increase cell cycle-related proteins in cancer. Earlier studies confirm that its proto-oncogenic role can be through multiple pathways other than p53 [106].

### 4.5. Liver Cancer

The sixth most frequently detected cancer worldwide is Liver cancer. Most liver cancers (approx. 75–-90%) are primarily hepatocellular carcinomas, malignant tumors in the liver’s parenchymal cells. The incidence of hepatocellular carcinomas is associated with chronic hepatitis B virus & hepatitis C virus infection and other factors like alcohol-related cirrhosis, smoking, etc. The other liver cancer type is intrahepatic cholangiocarcinoma, a tumor in cells lining bile ducts and can occur due to the high prevalence of chronic liver fluke infestation [163]. In hepatocellular carcinomas, the aberrant Wnt signaling and abnormal increases in β-catenin regulated by disheveled (Dsh/Dvl) mediators are responsible for tumor growth. Recently, Prickle-1 as a Dvl-associated protein has been identified in human hepatocellular carcinomas cells. A novel mechanism associated with Prickle-1 and Dvl3 in the Wnt/β-catenin pathway shows that Prickle-1 facilitates the degradation of Dvl3 via the ubiquitination pathway, thereby suppressing β-catenin activity and cell growth. Therefore, the under expressed Prickle-1 results in the accumulation of Dvl3 and β-catenin and larger tumor growth [108]. TRIM proteins act as an E3 ubiquitin ligase; also, the role of TRIM31 has been demonstrated lately. Its upstream expression is responsible for malignant behavior in hepatocellular carcinoma cells via the nTORC1 pathway. TRIM31 interacts with Tuberous sclerosis complex1 and Tuberous sclerosis complex 2 complex (an upstream suppressor for mTORC1 pathway) and mediates E3 ligase linked ubiquitination and degradation of the interacted complex [109].

Similarly, TRIM7 has shown its correlation with proto-oncogene Src, the nonreceptor tyrosine kinase family in a clinical specimen. The activation of Src triggers multiple cellular cascades PI3K and protein kinase B (PKB), mitogen-activated protein kinase, and signal transducer and activator of transcription 3, which are vital for cell survival. Therefore, TRIM7 and Src negative interrelation affect the course of action regarding hepatocarcinoma cell progression [110]. TRIM32 acts as an oncogene by downregulating the activity of apoptosis, cell cycle arrest, and senescence occurring due to stress in the first place. TRIM32 overexpression mediates p53-dependent activity to seize and promotes oncogenic transformation in p53-linked responses [111]. Yang, Y.F. et al., reported the oncogenic activity of TRIM65, which is regulated by HMGA1. Collectively, it exerts ubiquitylation of Axin1, which in turn activates and accumulates β-catenin in the cell [112]. In hepatocellular carcinoma samples, the upregulation of UBE2L3 is also suspected to be the reason for tumor progression. The raised UBE2L3 mediates the glyco3β degradation via proteasome-degradation, which blocks the activation of p65 in cellular signaling. The upregulated UBE2L3 activity is seen as a critical pro-tumorigenic factor in liver cancer [113]. UBE2T is generally aimed by miR-543, which is, however, low in hepatocellular carcinoma conditions. The UBE2T ectopic expression results in lowering p53, p21, and noxa facilitated by the ubiquitination and degradation of p53, overall, suggesting the role of UBE2T as a prognostic factor in hepatocellular carcinomas [114].

**Figure 6:**
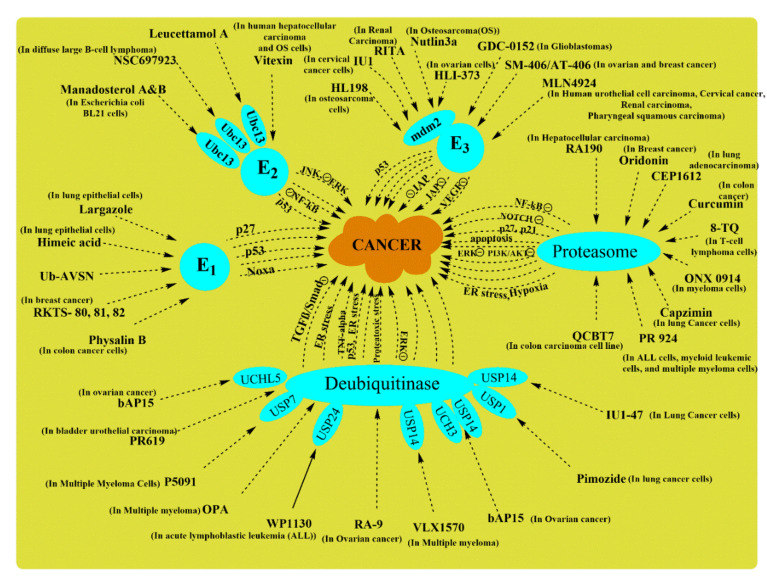
This figure depicts the complex interconnection between various enzymes and the pathway enzymes follow. The enzymes modulate one or more oncogenic pathways through components actively functional in the process.

Some of the deubiquitinases play a novel role in average cell growth, and their irregulating factors lead to cell masses. A K-63 linkage-specific deubiquitinase, CYLD, is a necessary modifier of NF-κB signaling and controls the ubiquitination state of NF-κB activation factor NEMO. NF-κB is known to be a critical controlling molecule for apoptosis. As CYLD functions as a tumor suppressor, its downregulation leads to the degradation of IκB and activation of NF-κB, resulting in apoptotic resistance in tumor cells [115]. Deubiquitinase enzyme “UCHL1” presented a contradicting action in tumor cells, having oncogenic and suppressor effects. In hepatocellular carcinoma, the UCHL1 gene is related to the apoptosis feature in tumor cells. However, the overexpressed c-myc in tumor cells was observed to have aggressive tumor growth due to UCHL1 that caused apoptotic resistance in hepatocarcinoma cells [116,117]. A very common ligase, NEDD4 overexpression, is associated with tumor onset and hepatocellular carcinoma progression. It is suggested that NEDD4 oncogenic activity is due to its influence on the activation of the PTEN/PI3K/AKT signaling pathway as NEDD4 depletion affects the phosphorylation of AKT (Figure 6) [118]. A member of the ubiquitin-like modifier family, deubiquitin FAT10, was identified as the most upregulated gene in lung cancer. Yuan, R., et al., have reported the upregulation of FAT10 expression in 90% of hepatocellular carcinoma patients, though the exact mechanism is yet to be identified [119].

### 4.6. Cervical Cancer

Cervical cancer is the fourth most known female malignancy in the world, and approximately 90% of cervical cancer occur in low-income and middle-income countries where organized screening and HPV vaccination programs are lagging [164]. Tobacco smoking was found to be a paramount causative factor for cervical precancer and cancer in a cohort study on more than 300,000 women [165]. The most common histological subtypes are squamous cell carcinoma and adenocarcinoma that account for approximately 70% and 25% of all cervical cancers, respectively [164]. DNA damage due to internal and external factors results in DNA double-stranded break can give rise to an imbalanced cell process. The key to recognition, signaling, and repair of DNA (double-stranded break) is the MRE11-RAD50-NBS1 (MRN) complex and mediator of MDC1 (DNA damage checkpoint protein 1). USP7 interacts with the MRN-MDC1 complex and stabilizes MDC1. The accumulated complex leads to the recruitment of ub p53 binding protein 1 and BRCA1 (breast cancer protein 1) to DNA double-stranded break [120]. E2-EPF is a member of ubiquitin-conjugating enzymes and is overexpressed in cervical squamous cancer through its effect on the pVHL-HIF pathway. Hypoxia predisposes to high tumor metastasis by inducing hypoxia-inducible factors (HIF-1).

Studies have suggested E2-EPF role in stabilizing HIF-1α via selectively targeting pVHL in the normoxic situation, and the forced expression of E2-EPF speeds up tumor proliferation, metastasis, and invasion [121]. E3 ligase MARCH 7 acts as a tumor-promoting gene in human cervix cancer via interacting with VAV2, triggering the activation of CDC42 and RAC1, i.e., VAV1/RAC1/CDC42 pathway [122]. The low expression of “ovarian-tumor proteases deubiquitinase 5” is connected with metastatic nodes, tumor stages, and tumor subtypes, such as those associated with PI3K-AKT signaling, epithelial-mesenchymal transition, and hormones. The tumor activity of “ovarian-tumor proteases deubiquitinase 5” is also observed in cervical cancer and can be related to the same signaling pathways, one or all, as mentioned above [123]. Ubiquitin-like, containing PHD and RING finger domains 1, UHRF1 regulates the UBE2L6 (conjugating enzyme) gene, and the normal UHRF1 function is to restore the UbcH8-induced apoptosis. There is an increased level of UHRF1, which regulates UBE2L6 by promoting hypermethylation in cervix cancer cells [124]. In a study, the enzyme USP18 is observed to act as a promoter of cell proliferation and inhibitor of apoptosis in cervical cancer cells. It might have pro-proliferative and anti-apoptotic factors by regulating PI3K/AKT pathway as USP18 knockdown suppressed AKT phosphorylation in tumor cells [125]. Mechanistically, UBE3A function has been demonstrated in in vitro cells through its binding members, HPV18 E6 and E6 target protein p53, and the loss of either of them blocks the effect of UBE3A. The reduction in UBE3A increased ERK pathway signaling and a decrease in growth factor-mediated ERK activation. Therefore, UBE3A negates the activated p53 consequences on ERK signaling pathway [126]. The USP8 protein level is higher in cervical squamous carcinoma cells. It is believed that USP8 directly deubiquitylates and stabilizes the long isoform of FLICE like inhibitory protein (FLIPL) in the cervical cancer cell line. The final effect of FLIPL can be stated as a cell death receptor mediating cell apoptosis. So, one can conclude that the USP8-overexpressing cells have suppressed the apoptotic pathway. However, previous studies on USP8 suggest its tumor effect via stabilizing EGFR signaling pathway [127].

TRIM 24 has a positive role in the progression of growth in tumor cells of various cancers. The accumulated TRIM24 activates the NF-κB/AKT pathway and thereby regulates cyclin D activity in the cell cycle in cervical cancer cell lines [128]. Cell cycle proteins are also regulated by TRIM59 ligase leading to tumor progression and growth. However, studies depict its inculcating nature in the p53 pathway and the activation of Ras/Rad, which triggers an oncogenic protein ERK signaling pathway. Knockdown of TRIM59 in cells inhibited cell progression by causing cell cycle arrest at phase S, and both the mentioned pathways are associated with the cell cycle arrest [129]. Exceptionally, a few TRIM members like TRIM3 show opposite activity towards growth property in tumor cells. TRIM3 actively enhances Caspase 3 and p53 activity and negatively affects the p38 pathway, responsible for cervical cancer [130].

### 4.7. Leukemia

Leukemia is a malignancy (cancer) of blood cells, and there are two types—acute and chronic; further distinguished based on cells. Acute myeloid leukemia is characterized by an increase in myeloid cells in the marrow and maturation stage arrest. The US’s annual incidence is approximately 2.4 per 100,000, which increases progressively with age having 5 years life expectancy of less than 15% [166]. Acute lymphoblastic leukemia encompasses a group of lymphoid neoplasms that morphologically and immunophenotypically are similar to B-lineage and T-lineage precursor cells representing 75% of acute leukemias [167]. Chronic myeloid leukemia is associated with a specific genetic lesion, the Philadelphia chromosome, which harbors BCR-ABL oncogene [168]. In leukemia cells, the increased mass of ubiquitinated protein is certainly not due to imbalanced degradation of ubiquitinated protein but because of elevated activity in the ubiquitination pathway [169]. The UBA2-WTIP fusion gene is suggested to be an oncogenic fusion gene that contains the N-terminus E1 enzyme member, VAE ubiquitin-like domains of UBA2, and the C-terminus LIM domains of WTIP. Mechanistically, the UBA2-WTIP fusion induces phosphorylation of ERK1/2, STAT3, and STAT5 and repels WTIP-induced mammalian processing body formation [131].

UBE2Q2 and CDC34 are E2 enzymes; the involvement of the components of the ubiquitin-proteasome pathway in leukemia has been known in studies. UBE2Q2 activity is significantly upregulated in acute lymphoblastic leukemia compared to normal tissue. Additionally, part of the pathway remains unknown [132]. CDC34 is also overexpressed in pediatric acute lymphoblastic leukemia. CDC34 is known to be a cell cycle regulator by its complex form with SCF and degrades IκB protein; therefore, hypothetically, this can cause CDC34 activity [170]. The UBE2E1 expression is adversely related to acute myeloid leukemia lifespan, though its role is unclear. UBE2E1 links with HOX gene regulation for its prognostic role in acute myeloid leukemia because HOX gene (HOXA9 and HOXA10) promotes acute myeloid leukemia leukemogenesis [133]. Expression of Fbxw7, a subunit of SCF ubiquitin ligase complex, is essential for regulating the threshold of c-Myc in favor of leukemia-initiating cells in chronic myeloid leukemia. The mutated Fbxw7 can activate c-Myc, a protein thought to be connected with Notch1, which, in turn, activates a group of genes necessary for the transformation to leukemia [134]. ARIH2 gene encodes an anti-proliferative E3 ubiquitin ligase, Triad1, which has a role in leukemogenesis, is induced by M11-E11 (MLL1 fusion protein) in acute myeloid leukemia. The presence of HOX genes affects the expression of Triad1 in M11-E11+ cells; therefore, Triad1 activity is regulated by HOX genes [135]. The histone H2B E3 ligase RNF20 is an additional chromatin regulator necessary for mixed-lineage leukemia fusion mediated leukemogenesis. When the transcriptional regulators are disrupted, mixed-lineage leukemia-fusion proteins modify gene expression in hematopoietic cells via interacting with histone H3 lys79 (H3K79) methyltransferase DOT1L. To balance the local levels of H3K79 methylation by DOT1L, RNF20 is required [136]. A role of deubiquitinase, USP7, is observed to be upregulated in human T-acute lymphoblastic leukemia cell lines and patient samples. USP7 can stabilize NOTCH1 protein level in in vitro and in vivo records, and the interaction is supported by the ubiquitin-like and MATH domains of USP7 [137].

An in vivo study was conducted for an enzyme USP22 to identify its response in the leukemia model. It was observed that USP22 promotes glioma tumorigenesis through deubiquitinating BMI1, which serves to trigger its oncogenic action in cells [138]. NOTCH and β-catenin signaling, additionally with an increase in glycogen synthase kinase 3β pathway, can lower the expression of ligase enzyme TRIM62. Loss of TRIM62 showed progression in tumorigenesis in cells due to various oncogenic proteins in cells. A low level of p53 and a high level of mdm2 also affect the functioning of TRIM62 in cells [139]. Multiple enzymes in different types of cancer are described above in the review. However, it is not right to say if the ubiquitin-proteasome pathway only exhibits an oncogenic effect in the cell. These enzymes are present in abundance in every part of the body and have numerous effects. They act upon multiple pathways, but these enzymes’ central pathway to give an oncogenic effect is considered the mode of action. These enzymes regulate the proper functioning of cellular activity via targeting and degrading abnormal proteins in normal conditions. The modified transcriptional factors in these enzymes give rise to their altered expression, and the above collected data reveals how these modifications progress into cancer. Exploring ubiquitin-proteasome enzymes have a pursuit towards developmental in cancer studies.

## 5. Preclinical Studies

### 5.1. In Vivo Studies

Various studies have been done on ubiquitin-proteasome inhibitors, which support the suppression of tumor factors in different signaling pathways. In the given table, preclinical in vitro and in vivo studies of various inhibitors in different cancers are discussed (Table 2).

### 5.2. In Vitro Studies

Preclinical studies start with understanding the impact of the drug on cell lines. Due to these cell line cultures, most animal lives are spared to understand its toxicity on body cells (Table 3).

## 6. Clinical Trial Drugs with Ongoing Stage and Category

There are several drugs that are conducted in a better way to prevent cancer. Lists of such drugs namely TAK-243, Disulfiram and Cooper, KPG-818, Vorinostat and Bortizomib, MLN4924, Bortezomib + Doxorubicin, NPI-0052, NPI-0052 + Vorinostat, JNJ-26854165, Bbortezomib (PS-341), TAK-981 + Pembrolizumab, Oprozomib, Carfilzomib, MLN9708, MLN9708 + Vorinostat, GSK2110183, Trastuzumab and PS-341, Finasteride have been summarized here in this Table 4.

## 7. Cancer Therapeutic Strategy via Targeting UPS

Instability in signaling pathways and their components can cause dysregulation in many intracellular processes that result in malignancies since ubiquitin-proteasome systems are crucial to almost every physiological function in an organism. A breakdown in their signaling results in serious diseases [211]. Ubiquitin proteasome system regulates the cell cycle, and abnormalities in its activity lead to oncogenesis as overexpression or down-regulation of ubiquitin-proteasome system components can lead to critical cellular phenotypes. Various studies on ubiquitin-proteasome systems have recently been published, suggesting its involvement in cellular function and the possibility of targeting ubiquitin-proteasome system components for novel anti-cancer agents [212]. Many discoveries have come across drugs showing inhibitory action on a particular enzyme/proteasome for a potential neoplastic agent. Substantial evidence on the relationship between enzymes with their signaling pathway has been proved. Therefore, it will ultimately suppress its role in the pathway if the enzyme is inhibited, let it be oncogenic or non-oncogenic. In this section of the review, drugs with inhibitory action on the ubiquitin-proteasome system have been discussed with their effect on the connected signaling pathway.

### 7.1. E1 Ubiquitin-Activating Enzyme Inhibitors

**Figure 7:**
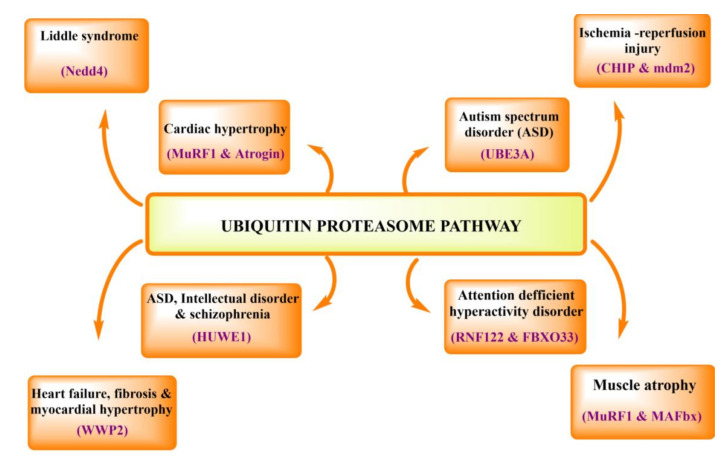
This figure represents the UPP and their respective enzymes i.e., Nedd4, MuRF1& Atrogin, UBE3A, CHIP & mdm2, HUWE1, RNF122 & FBXO33, WWWP2, MuRF1 & MAFbx IN Different diseases.

Ubiquitination is an essential modification system for various cellular proteins. The first step in the ubiquitin conjugation system is ubiquitin’s activation to high energy intermediate, catalyzed by E1 (the ubiquitin-activating enzyme). The core of the ubiquitin-proteasome system will halt by simply inhibiting the E1 enzyme. Yang, Y. et al., discovered a drug with the chemical formula “4[4-(5-nitro-furan-2- yl-methylene)-3,5-dioxo-pyrazolidin-1-yl]-benzoic acid ethyl ester” and named it PYR-41 (Figure 7). It was examined in vitro to know its focus on the particular enzyme when it suppressed ubiquitination function and reported it as a first cell-permeable E1 inhibitor. The pyrazone derivative PYR-41 weakens the activation of NF-κB activation through enhancing IκB activation. PYR-41 also prevents the degradation of p53, which gives a transcriptional signal for cell death.

Moreover, this drug has the potential to kill mutated p53-expressing cells as well [213]. The research reported Largazole and its analog selectively inhibit E1 activity in vitro by blocking the adenylation-activation step without disturbing ubiquitin transfer from E1 to E2. Largazole in cell culture affects multiple pathways other than inhibiting activating enzymes as it also inhibits proteasome activity. It is involved in preventing the degradation of an essential anti-oncogenic factor, p53, and inhibits cdk activity, enhancing p53 efforts in cell death [191].

In cell lines and primary samples of acute myeloid leukemia, TAK-243 can trigger cell death and downregulate clonogenic growth. Barghout, S.H. et al., evaluated a first-in-class UBA1 inhibitor TAK-243 in acute myeloid leukemia preclinical models and also supported TAK-243 for a clinical trial in their patients. In acute myeloid leukemia cells, TAK-243 was seen to attach preferentially with UBA1 over the similar enzymes, UBA2, UBA3, and UBA6 [214]. TAK-243 decreases the level of ubiquitin-protein conjugates and stabilizes short-lived proteins such as p53, myeloid cell leukemia-1, c-Myc, etc. Notably, TAK-243 has overcome resistance to conventional drugs, including bortezomib and carfilzomib resistance, in cell-line models. It also showed effectiveness against primary cells from refractory/relapsed myeloma patients [215]. TAK-243 is also known under names pevonedistat, MLN7243, and MLN4924 in Phase I/II clinical trials [216]. Another Uba1 inhibitor, PYZD-4409, is structurally similar to PYR-41 and induces cell death predominantly in primary patient samples and hematological cell lines over normal hematopoietic cells. PYZD induced cell death is associated with endoplasmic reticulum stress and stabilization of cyclin D3 along with p53. It is important to note that the intraperitoneal injection of PYZD-4409 in leukemic mouse decreased the tumor weight efficiently while comparing its untoward side effects [169,217,218]. Panepophenanthrin has been claimed in research that it is the first inhibitor of the ubiquitination pathway extracted from mushroom strain IFO8994 [219]. It belongs to an epoxyquinoid class of natural products; its unique structure shows its potential for oxygen functionalization. Future research might provide more information on its mechanistic approach in showing anti-oncogenic effects [220]. A new strain was found while investigating panepophenanthrin for its biological derivatives responsible for its impact on ubiquitination mechanism in vivo and in vitro. Matsuzawa, M., 2006 discovered new derivatives, RKTS-80, -81, and -82, as E1 inhibitors when tested on human breast cancer MCF-7 cells which efficiently in a dose-dependent manner blocked the cell growth. Unfortunately, other than being cell-permeable, no other property for these new derivatives is known [202].

### 7.2. E2 Ubiquitin-Conjugating Enzyme Inhibitors

After the ATP-dependent activation of ubiquitin via the ubiquitin-activating enzyme, ubiquitin is next transferred to a specific cysteine residue on any of the various ubiquitin-conjugating enzymes called E2 proteins. Therefore, if this component of the ubiquitin-proteasome system is inhibited, it will ultimately obstruct further process steps. Previously, a drug Bay 11-7082 was reported to have an inhibitory activity on NF-κB and induces apoptosis in human T-cell lymphotropic virus-induced negative T-cells by downregulating the antiapoptotic gene (Bcl-xL) expression [221]. But in recent studies, it has been reported that Bay 11-7082 does not affect NF-κB; instead, it inhibits Ubc 12 & UbcH7 and suppresses the activation of LPS (lipopolysaccharide)-stimulated RAW macrophages, with another inflammatory pathway [222]. As lipopolysaccharides induce activation of the noncanonical NF-κB signaling pathway, Bay 11-7082 can show an anti-tumor role in cancer via inhibiting Ubc 12 (have a role in ovarian cancer) and UbcH7 (DSB regulator). NSC697923 is a UBE2N inhibitor that can exhibit a cytotoxic effect on neuroblastoma cell lines, evidenced by its ability to induce p53-induced apoptosis. Even in mutant p53 cells, NSC697923 has shown anti-tumor response via activating the JNK pathway [223]. In diffuse large B-cell lymphoma, NSC697923 impeded the activity of Ubc13-Uev1A as it suppresses NF-κB signaling activity inactivated and germinal center B-cell-like large diffuse B-cell lymphoma cells [198].

CC0651 is a reversible small-molecule inhibitor of Ube2R1, and molecular studies revealed its binding with allosteric mode (away from the active site). Some E2 families use its backside (surface opposite to catalytic site) interaction to enhance, inhibit or modulate catalytic activity [224]. CC0651 has been identified as an inhibitor of the human CDC34 ubiquitin-conjugating enzyme. The inhibitory activity of CC0651 is concerned with its interference in the step “removal of Ub” to acceptor Lys residues; however, it plays no part in interaction with E1 or E3 or in Ub thioester bond formation [225,226]. TZ9 (Twelve triazines) and 4-amino- N ′-phenyl-6-(arylamino)-1,3,5-triazine-2-carbohydrazides are reported to be Rad6B- inhibitor as diaminotriazinylmethyl benzoate anti-cancer agents. Similar to these, series of N′-phenyl-4,6- bis(arylamino)-1,3,5-triazine-2-carbohydrazides (6a–e) and 4-amino-6-(arylamino)-1,3,5-triazine-2-carbohydrazides (3a–e) have been synthesized and abbreviated as ‘new triazines.’ In the absence of E3 ligases, Rad6B can ubiquitinate not only histones but also ubiquitinate β-catenin. A ubiquitinated β-catenin is insensitive to 26S proteasome, indicating that Rad6B is essential for β-catenin stabilization and activation in breast cancer [227]. These new triazines inhibit Rad6B ubiquitin conjugation and have shown anticancer action against many human cancer cell lines [228].

### 7.3. E3 Ubiquitin Ligase Inhibitors

When the polyubiquitinated chain is formed in the ubiquitination process, the chain is attached with substrate protein with E3 ligase. E3 ubiquitin ligase transfers substrate protein on the ubiquitin chain for the attachment, which takes it to the proteasome; thus, E3 enzyme inhibitors may protect the protein from degradation. Gene p53 acts as a tumor suppressor, but its interaction with ligases mdm2 suppresses p53 activity, resulting in tumor formation. This interaction between mdm2 with p53 can be targeted for a therapeutic approach, and one of the selective E3 ligase targeting molecules is called Nutlins. This cis-imidazoline analog interacts with the p53 binding site instead of mdm2, leading to p53 stabilization, cell cycle arrest, and ultimately apoptosis in cancer cells [229]. A highly potent drug, RG7112, is a small molecule inhibitor that selectively targets structures found within the p53 binding site of mdm2 [230]. Cullin-RING ubiquitin ligases (CRLs) are the most prominent family of E3 ligases, and it requires cullin neddylation for their activation.

Ligase NEDD activity has been observed in various cancers mentioned above. The NEDD8- activating enzyme is inhibited by the MLN4924 drug, which blocks cullin neddylation and, therefore, inactivates CRLs and promotes apoptosis. In ovarian cell lines, reducing CRL4 components (Roc1/2, DDB1, and Cul4a) has also shown an inhibitory effect on tumor cells, similar to the MLN4924 effect. Therefore, the tumor-suppressing effect of CRL4 is suggested to contribute to the chemotherapeutic effect of MLN4924 in ovarian tumor cells [231]. The mdm2 protein contains a C-terminal RING domain which coordinates two Zn atoms responsible for p53 nuclear export and degradation via proteasome machinery. In colon and breast cancer cells, the critical role of Zn was exhibited in the reactivation of p53 when MI-219 ((3-(4,5-dimethylthiazol-2-yl)-2,5-diphenyltetrazolium bromide (MTT)) was used. However, the activity of mdm2 inhibitor (apoptosis and colony formation), namely, MI-219, was enhanced under the influence of ZnCl2 [232]. A small-molecule TAME (tosyl-L-arginine methyl ester), belonging to a microtubule inhibitor, induces mitotic arrest and inhibits the ubiquitin ligase function of APC, i.e., This anaphase-promoting complex is usually required for mitotic exit. The mitotic arrest and anaphase-promoting complex inhibition are a response to the activation of the spindle assembly checkpoint. It is given in the form of a cell-permeable derivative- proTAME. The antagonism action of both the factors, i.e., anaphase-promoting complex, and spindle assembly checkpoint, is responsible for amplifying TAME’s influence on cells [233]. Mdm2 ligase activity is also down-regulated by treating a small molecule inhibitor drug (JNJ-26854164) as the influence of mdm2 is reversed on p53, which induces apoptosis in cells [234]. Other than p53 mediated antitumor activity of mdm2 also influences pathways like TGF-β.

In ovarian cells, E3 ligase inhibitor (HLI-373) targets the C-terminus of mdm2 and impairs TGF-β promoted epithelial-mesenchymal transition via β-smad-snail/slug pathway [204,235]. Thalidomide was previously used in morning sickness and is nowadays used for treating multiple myelomas. The primary target of thalidomide is cereblon which forms an E3 ligase complex with disrupted DNA-binding protein 1 and cullin-4A. The drug selectively binds with cereblon, thereby, inhibit its associated ubiquitin ligase activity [236,237]. Another mdm2 antagonist, MI-312 with cisplatin combined treatment, act synergistically, thereby suppressing cell growth and inducing apoptosis. The combination activates pathways that are downstream of p73 involving the necessary cell cycle modulator p21WAF1, showing its p53-independent mode of action [238].

## 8. Proteasome Inhibitor

Ubiquitination is found to target proteins for proteasomal degradation explained above with sequence of action and its composition. The proteasome is believed to have different subunits in its 20S core particle: caspase-like, trypsin-like, chymotrypsin-like [239]. Bortezomib which is widely used for treating multiple myeloma is known to have proteasome inhibiting activity. Other commonly used proteasome inhibitors are ixazomib and carfilzomib. Recent reports suggest that their concentration highly influences their activity at low concentrations. These inhibitors affect the chymotrypsin-like proteasome activity, inhibiting caspase-like activity at high concentrations [240,241]. Through extensive research with their known properties, they can be used against various cancer growths. In in vitro research on the cytotoxic effects of epoxomicin, it was found that it was a potent inhibitor of the proteasome’s chymotrypsin-like activity. Previously, it was suggested to have an inhibitory effect on NF-κB activation in cancerous cell line culture [242]. However, in amelanotic melanoma cells, it exhibits anti-apoptotic activity in the mitochondrial pathway by accumulating the noxa proapoptotic factor and anti-apoptotic Mcl-1. Epoxomicin inactivates caspase, which causes an increase in cdk inhibitors, i.e., p21Cip1/Waf1 and p27Kip [243].

The upregulated levels of cdk inhibitors are also seen in leukemia cell lines when treated with a second-generation drug, carfilzomib, for hematological malignancies. Carfilzomib is a tetrapeptide epoxyketone proteasome inhibitor, and extensive research has shown that it induces cell cycle arrest in the G2/M phase by inhibiting cyclin-dependent kinase 1 [244]. A proteasome inhibitor delanzomib (otherwise called CEP-18770), has shown promising anti-myeloma property in preclinical studies. It is a reversible P2 threonine boronic acid inhibitor, binds with β5/β1 proteasome subunits inhibiting chymotrypsin-like (β5) and caspase-like (β1) activity of proteasome [245]. A new proteasome inhibitor, QCBT7 (aquinolin-chlorobenzothioate 7), showed its cytotoxic effect in multiple cancer cell lines. It is a stable derivative of quinoline-8-thiol, which attacks the regulatory subunit instead of targeting the proteasome’s catalytic subunit. Overall, its anti-tumor activity is manifested by inducing proteasome inhibition, endoplasmic reticulum stress, hypoxic response, and glycolysis, resulting in cell death. A potential biomarker of proteasome inhibitor was discovered in the same study named PFKPB4 (6-phosphofructo-2-kinase/fructose-2,6-biphosphatase 4) can be used for monitoring drugs therapeutic response in pancreatic cancer [208]. A novel proteasome inhibitor, physalin B, was isolated as interfering in the ubiquitin-proteasome pathway. However, when more information was obtained, physalin B impairs proteasome function in the ubiquitin-proteasome pathway. However, it was found to impair proteasome function in the ubiquitin-proteasome pathway when more information was obtained. It promotes apoptosis in a cell by inducing pro-apoptotic NOXA, a feature of the ubiquitin-proteasome pathway [203].

## 9. Conclusions and Future Perspective

Ubiquitin proteasome pathway regulates uncountable body functioning through its network of enzymes spread all over the body. It is connected like a thread sewed in a cloth with many transduction process pathways, including cellular signalling pathways for cell differentiation and division. Therefore, UPP’s role in diseases like cancers is explained in various researches, and the disrupted expression of specific enzymes of the pathway responsible for cancer needs immense study. For example, the enzyme UBE2T in colorectal cancer has shown its oncogenic role in cells. It has shown its relation with p53, pentose phosphate pathway, and other pathways, but its exact role in the pathway is still unknown. However, if this enzyme is studied in other cancer types, it might indicate its pathways. Molecular mechanisms associated with metabolic reprogramming in cancer have been studied extensively over the last few decades. The roles of ubiquitination and deubiquitination as cancer modulators are highlighted in this review.

As discussed in the review, deubiquitinase enzymes also have a characteristic property of preventing protein degradation by proteasome via attaching with the substrate protein and blocking its link with E3 ligase. In the process, it might save a pro-oncogenic protein like UCHL1, which has been observed to be over-expressed in the hepatocarcinoma state. The connection between these enzymes and cancer can be used as a therapeutic strategy. Some inhibitors of enzymes like ligase inhibitors, deubiquitinase inhibitors, proteasome inhibitors are already under clinical trial. It might result in offering a suitable drug for curing this dreadful disease. In clinical trials, combination treatments are being encouraged like bortezomib + doxorubicin for a successful synergistic approach against cancer cells. Overall, due to the vast availability and interlink of these UPP enzymes involved in oncogenic activity, they can be targeted for a favorable outcome to treat cancer. To summarise, if the mechanism of UPP dysfunction and the precise function of enzymes are fully explored, it may pave the way for treating a variety of diseases other than cancer. Drugs like vitexin (a platelet aggregation inhibitor) efficiency have been checked on multiple cancer cultures but have no details about its effect on animals.

## Figures and Tables

**Table 1:** The table summarizes various physiological roles of UPP and the enzymes/proteins involved in a cancer type.

S. No.	Type of Enzyme	Enzymes Involved	Modulation of Pathways Involved	Cancer Type	References
1.	E3 ligase	Ectodermin, TRIM 47	↓ Smad 4 in TGF-β signaling	Promotes colorectal cancer	[75]
2.	E3 ligase	FBXW7	↓ Wnt/β-catenin	Inhibits colorectal cancer	[71]
3.	Deubiquitinating enzymes	USP5	↑ Wnt/β-catenin	Promotes colorectal cancer	[72]
4.	Deubiquitinating enzymes	USP11, USP21	↑ERK/MAPK	Promotes colorectal cancer	[73,74]
5.	Deubiquitinating enzymes	UBE2T	↓ p53 pathway, ↑pentose phosphate pathway, etc.,	Promotes colorectal cancer	[76]
6.	E3 ligase	TRIM 67	↓ p53 degradation	Inhibits colorectal cancer	[75]
7.	Ubiquitin-conjugating enzyme E2C	UBE2C	Pro-apoptotic	Inhibits esophageal cancer	[77]
8.	E3 ligase	TRIM36	↑ Wnt/β-catenin	Promotes esophageal cancer	[78]
9.	E3 ligase	TRIM44	↑ mTOR	Promotes esophageal cancer	[79]
10.	E3 ligase	TRIM16	↑ TGF β/Snail	Promotes esophageal cancer	[80]
11.	E3 ligase	RNF113A	----	Promotes esophageal cancer	[81]
12.	E3 ligase	MARCH 8	----	Promotes esophageal cancer	[82]
13.	E3 ligase	Gankyrin	↑ p53 degradation	Promotes esophageal cancer	[83]
14.	Deubiquitinating enzymes	Ubiquitin carboxyl-terminal hydrolase 37	↑ TGF-β signaling	Promotes esophageal cancer	[84]
15.	E2 ligase	Uev1A	bone morphogenetic protein signaling	Inhibits osteosarcoma	[85]
16.	E3 ligase	Nedd4	↑ TGF-β signaling	Promotes osteosarcoma	[86]
17.	E3 ligase	USP7	↑ Wnt/β-catenin	Promotes osteosarcoma	[87]
18.	Deubiquitinating enzymes	USP1	stabilize “inhibitors of DNA binding.”	Promotes osteosarcoma	[88]
19.	E3 ligase	Deltex1	↓ NOTCH/HES1	Inhibits osteosarcoma	[89]
20.	E2 ligase	FAT10	↓ Hippo/YAP1	Inhibits osteosarcoma	[90]
21.	Deubiquitinating enzymes	USP39	↑ p21	Inhibits osteosarcoma	[91]
22.	E3 ligases	TRIM46, TRIM21, TRIM14, and TRIM23	↑NF-κB	Promotes osteosarcoma	[92]
23.	E3 ligases	TRIM59 and TRIM7	↓ p53 and E-Cadherin	Promotes osteosarcoma	[93,94]
24.	Deubiquitinating enzymes and E2 ligases	USP22 and UBE2T	↑ PI3K/AKT	Promotes osteosarcoma	[95,96]
25.	E3 ligases	Smurf1	↑ TGF-β signaling	Promotes lung cancer	[70]
26.	E3 ligases	NEDD4-1	↑ PI3K/PTEN	Promotes lung cancer	[97]
27.	E3 ligases	NEDD4	EGFR mutation	Promotes lung cancer	[98]
28.	E3 ligases	UBE3A	↓ p16INK4a	Promotes lung cancer	[99]
29.	E3 ligases	HRD1	↓ Sirtuin 2	Promotes lung cancer	[100]
30.	Deubiquitinating enzymes	USP37	↑ c-Myc	Promotes lung cancer	[101]
31.	E3 ligases	TRIM7, TRIM71	↑ NF-κB	Promotes lung cancer	[102,103]
32.	E2C ligases	UBE2C	↑ ERK	Promotes Lung cancer	[104]
33.	E3 ligases	UBE3C, TRIM59	↓ p53	Promotes Lung cancer	[105,106]
34.	Deubiquitinating enzymes	USP22	↑ ERK/AKT	Promotes lung cancer	[107]
35.	E3 ligases	Prickle-1	↓ Wnt/β-catenin	Inhibits liver cancer	[108]
36.	E3 ligases	TRIM31	↑ mTOR	Promotes liver cancer	[109]
37.	E3 ligases	TRIM7	↓ PI3K	Inhibits liver cancer	[110]
38.	E3 ligases	TRIM32	↑ mutated p53	Promotes liver cancer	[111]
39.	E3 ligases	TRIM65	↑ β-catenin	Promotes liver cancer	[112]
40.	E2 ligases	UBE2L3	↓ p65	Promotes liver cancer	[113]
41.	E2 ligases	UBE2T	↓ p53, p21, and noxa	Promotes liver cancer	[114]
42.	Deubiquitinating enzymes	CYLD	↓ NF-κB	Inhibits liver cancer	[115]
43.	Deubiquitinating enzymes	UCHL1	Apoptotic resistance	Promotes liver cancer	[116,117]
44.	E3 ligases	NEDD4	↑ PTEN/PI3K/AKT	Promotes liver cancer	[118]
45.	E3 ligases	FAT10	-----	Promotes liver cancer	[119]
46.	E3 ligases	USP7	Facilitates DNA repair by stabilizing MDC1	Promotes cervical cancer	[120]
47.	E2 ligases	E2-EPF	↑ pVHL-HIF	Promotes cervical cancer	[121]
48.	E3 ligases	MARCH 7	↑ VAV1/RAC1/CDC42	Promotes cervical cancer	[122]
49.	Deubiquitinating enzymes	Ovarian-tumor proteases deubiquitinase 5	↑ PI3K-AKT	Promotes cervical cancer	[123]
50.	E3 ligases and E2 ligases	UHRF1, UBE2L6	Promotes hypermethylation	Promotes cervical cancer	[124]
51.	Deubiquitinating enzymes	USP18	↑ PI3K/AKT	Promotes cervical cancer	[125]
52.	E3 ligases	UBE3A	↓ ERK	Inhibits cervical cancer	[126]
53.	Deubiquitinating enzymes	USP8	Stabilizes FLIPL and EGFR signaling	Promotes cervical cancer	[127]
54.	E3 ligases	TRIM 24	↑NF-κB/AKT	Promotes cervical cancer	[128]
55.	E3 ligases	TRIM59	↓ p53 pathway, ↑ Ras/Rad, ↑ ERK	Promotes cervical cancer	[129]
56.	E3 ligases	TRIM3	↑ p53 pathway, ↑ Caspase 3	Inhibits cervical cancer	[130]
57.	E1 ligases	UBA2	↑ ERK1/2, STAT3, and STAT5	Promotes leukemia	[131]
58.	E2 ligases and E2R1	UBE2Q2 and CDC34	↓ IκB	Promotes leukemia	[132]
59.	E2 ligases	UBE2E1	↓ HOX gene (HOXA9 and HOXA10)	Inhibits leukemia	[133]
60.	E3 ligase	Fbxw7	↑ c-Myc, Notch1	Promotes leukemia	[134]
61.	E3 ligases	Triad1	↓HOX genes	Inhibits leukemia	[135]
62.	E3 ligases	RNF20	Interacts with histone H3 lys79 (H3K79) methyltransferase DOT1L	Promotes leukemia	[136]
63.	E3 ligase	USP7	↑ NOTCH1	Promotes leukemia	[137]
64.	Deubiquitinating enzymes	USP22	Stabilize BMI1	Promotes leukemia	[138]
65.	E3 ligases	TRIM62	↑ NOTCH and β-catenin signaling	Promotes leukemia	[139]

**Table 2:** In vivo studies of the ubiquitin inhibitors supporting the suppression of tumors.

S. No.	Drug	Cancer	Signaling Pathway	Animal Models	Reference
1.	RNF152	Colorectal cancer	It is inactivating mTORC1 to induce autophagy and apoptotic cell death.	Immunodeficient nude mice	[171]
2.	RITA (2,5-bis[5-hydroxymethyl2-thienyl] furan, NSC 652287)	Renal carcinoma	Block TP53–mdm2 complex and reactivation of p53 and Induction of Tumor cell Apoptosis	Mouse xenograft model	[172]
3.	RA-9	Ovarian cancer	Apoptosis and proteotoxic stress	Mice xenograft model	[173]
4.	WP1130	T-cell acute lymphoblastic leukemia	Induces apoptosis by accelerating the collapse of mitochondrial transmembrane potential via USP24-Mcl-1 axis	Tumor xenografts in NOD-SCID mice	[174]
5.	The bis-benzylidine piperidone RA190	Hepatocellular carcinoma	Nuclear factor κB (NF-κB) signaling	Male nude mice CAnN.Cg-Foxn1nu/CrlNarl	[175]
6.	O-phenanthroline (OPA)	Multiple myeloma	Caspase cascade and endoplasmic stress response signaling	Murine xenograft model of human MM	[176]
7.	Nutlin-3a	Osteosarcoma	Competitively binds the mdm2-p53 interacting site, activates p53 pathway	Human xenograft OS animal model with *SAOS-2,* *U2OS,* *MG63* cell lines in SCID mice	[177]
8.	GDC-0152	Glioblastomas	Antagonists of the inhibitor of IAPs Postponed tumor formation and slowed down tumor growth	U87MG- iRFP cell grafted mice	[178]
9.	SM-406/AT-406	Human cancer cell (ovarian and breast cancer)	Antagonizes XIAP BIR3 induces rapid degradation of cellular cIAP1 protein	SCID mice bearing MDA-MB-231 xenograft tumors	[179]
10.	Oridonin	Breast cancer	Tumor suppressive effect via inhibiting Notch receptors expression	Male BALB/C athymic nude mice	[180]
11.	MLN4924	Human urothelial cell carcinoma, cervical cancer, renal carcinoma, pharyngeal squamous carcinoma	Inhibits cell viability and induced apoptosis in HUVECs (human umbilical vascular endothelial cells)	Xenograft SCID mice	[181]
12.	P5091	Colorectal cancer	Elevated mRNA level of IFN-γ and TNF-α	Female BALB/c mice (CT26 xenograft model)	[182]
13.	bAP15	Ovarian cancer	Regulating TGF-β signaling, dephosphorylating Smad2, inducing apoptosis	Mice xenograft models of SKOV3	[183]
14.	PR-619	Bladder urothelial carcinoma (UC)	Suppression of the Bcl-2 level	Nude mice Xenograft Matrigel culture	[184]
15.	CEP1612 [phthalimide-(CH2)8CH-(cyclopentyl) CO-Arg(NO2)-Leu-H]	Human lung adenocarcinoma	Accumulation of p21WAF1 and p27KIP1, inducing apoptosis	A-549 tumor-bearing nude mice	[185]
16.	Curcumin	Human colon cancer	Inhibit the proteasome and induce apoptosis	HCT-116 tumor-bearing ICR SCID mice	[186]
17.	P5091	Multiple Myeloma Cells	Inhibited USP7 activity, decreased HDM2, and increased p21 levels, induces apoptosis	Human plasmacytoma xenograft and SCID-hu mouse models	[155]
18.	ECRG4	Esophageal cancer	Inhibits NF-κB expression and nuclear translocation, attenuates NF-κB target gene COX-2 expression	BALB/c nude mice	[187]
19.	8-(tosylamino) quinoline (8-TQ)	Human cancer cells	Inhibition of molecular signaling machineries composed of phosphoinositide 3-kinase (PI3K)/phosphoinositide-dependent kinase-1 (PDK1)/Akt and extracellular-signal-regulated kinase (ERK)	murine T-cell lymphoma RMA cells in mice	[188]
20.	VLX1570	Multiple myeloma	Decrease in ERK phosphorylation; USP14 inhibitor	Xenograft model in immunocompromised mice	[189]
21.	b-AP15	Large B cell lymphoma	Inhibits Wnt/β-catenin and TGFβ/Smad pathways; USP14 and UCHL5 deubiquitinases	Mouse xenograft models of SU-DHL-4 and SU-DHL-2 cells	[190]

**Table 3:** In vitro studies of the ubiquitin inhibitors supporting the suppression of tumors.

S. No.	Drugs	Category	Cell Lines	Reference
1.	Largazole	Ubiquitin activating enzyme (UAE) inhibitor	Kip16, a GFP-p27 expressing Cell Line	[191]
2.	Himeic acid A	UAE inhibitor	Western blotting with anti-Flag antibody	[192,193]
3.	Ub-vinylsulfonamide (Ub-AVSN)	UAE inhibitor	N597A variant and the WT assay	[194,195]
4.	Pimozide or GW7647	USP1/UAF1 inhibitor	H596 and H460 cell lines	[196]
5.	Leucettamol A	Ubc13-Uev1A inhibitor	Escherichia coli BL21 cells	[197]
6.	NSC697923	Ubc13-Uev1A inhibitor	ABC (activated B cell-like)-DLBCL cells and GCB (germinal center B cell-like)-DLBCL cells	[198]
7.	Manadosterols A and B	Ubc13-Uev1A inhibitor	Escherichia coli BL21 cells	[199]
8.	Vitexin	ubiquitin-conjugating enzyme E2-25K inhibitor	Rat pheochromocytoma PC12 cells, HepG2 (human hepatocellular carcinoma), and HOS (human osteosarcoma) cells	[200]
9.	HLI98 family (C, D, E)	Ubiquitin ligase enzyme inhibitor	SAOS cells	[201]
10.	RKTS-80, -81, -82	E1 inhibitors	human breast cancer MCF-7 cells	[202]
11.	Physalin B	proteasome inhibitors	human DLD-1 colon cancer cells	[203]
12.	HLI-373	E3 ligase inhibitor	ovarian SKOV3 cells	[204]
13.	ONX-0914	Immunoproteasome inhibitors	KMS-11 cells	[205]
14.	PR-924	Immunoproteasome inhibitor	Human T-cell ALL CCRF-CEM cells, human myeloid leukemic THP1 cells, and human multiple myeloma RPMI-8226 cells	[206]
15.	Capzimin	Proteasome inhibitor	HCT116 cell lines	[207]
16.	QCBT7	Proteasome inhibitor	colon carcinoma cell line HCT 116.	[208]
17.	IU1-47	USP14 inhibitor	A549 and H1299 cell lines	[209]
18.	IU1	USP14 selective inhibitor	HeLa and SiHa cells (cervical cancer cells)	[210]

**Table 4:** Clinical status of the drugs.

S. No.	Drug in Clinical Trials	Category	Cancer	Phase	NCT Number
1.	TAK-243 (formerly known as MLN7243)	UAE (ubiquitin-activating enzyme) inhibitor	Advanced Malignant Solid tumors	Phase I (terminated)	NCT02045095
2.	Disulfiram and Cooper	Zinc fingers and RING-finger ubiquitin E3 ligases inhibitors	Breast Neoplasm Female, Metastatic Breast Cancer	Phase 2	NCT03323346
3.	KPG-818	Ubiquitin ligase modulator	Selected hematological malignancies (multiple myeloma, mantle cell lymphoma, follicular lymphoma, diffuse large B-cell lymphoma, indolent lymphoma, adult T-cell leukemia-lymphoma, or chronic lymphocytic leukemia	Phase 1	NCT04283097
4.	Vorinostat (MK-0683) + Bortezomib	HDAC (Histone deacetylases) inhibitors + proteasome inhibitor	Multiple Myeloma	Phase 3	NCT00773747
5.	MLN4924	Nedd8 activating enzyme inhibitor	lymphoma or multiple myeloma	Phase 1 (completed)	NCT00722488
6.	Bortezomib + Doxorubicin	Proteasome inhibitor	Advanced, Recurrent, or Metastatic Adenoid Cystic Carcinoma of the Head and Neck	Phase 2 (completed)	NCT00077428
7.	NPI-0052	Proteasome inhibitor	Solid tumors, lymphomas, leukemias, and multiple myeloma.	Phase 1 (completed)	NCT00629473
8.	Marizomib(NPI-0052) + Vorinostat	Proteasome inhibitor + HDAC (Histone deacetylases) inhibitors	Non-Small Cell Lung cancer, Pancreatic cancer, Melanoma, Lymphoma Multiple Myeloma	Phase 1 (completed)	NCT00667082
9.	JNJ-26854165	E3 ligase inhibitors	Neoplasms	Phase 1 (completed)	NCT00676910
10.	Bortezomib (PS-341)	proteasome inhibitor	Squamous cell carcinomas of the head and neck (SCCHN)	Phase 1 (completed)	NCT00011778
11.	TAK-981 + Pembrolizumab	SUMOylation inhibitor + immunosuppressant	Advanced or Metastatic Solid tumors	Phase 1 Phase 2	NCT04381650
12.	Oprozomib	Proteasome inhibitor	Advanced Refractory or Recurrent Solid tumors	Phase 1 (completed)	NCT01129349
13.	Carfilzomib	Proteasome inhibitor	Neuroendocrine cancer	Phase 2	NCT02318784
14.	MLN9708	Proteasome inhibitor	Advanced non-hematologic malignancies	Phase 1 (completed)	NCT00830869
15.	MLN9708 + Vorinostat	Proteasome inhibitor + HDAC inhibitor	Advanced p53 Mutant malignancies	Phase 1	NCT02042989
16.	GSK2110183	Proteasome inhibitor	Multiple myeloma	Phase 1 (completed)	NCT01445587
17.	Trastuzumab + PS-341	Proteasome inhibitor	Breast cancer, Stage 4	Phase 1 (completed)	NCT00199212
18.	Finasteride	Ubiquitin-conjugating enzyme inhibitor	Adenocarcinoma of the ProstateStage II Prostate cancer	Phase 2	NCT00438464

## Data Availability

Not applicable, the study did not report any data.

## Abbreviations

BMP-2	Bone morphogenetic protein-2
BTRC	β-transducin repeat containing
Cdc20	Cell division cycle 20
DDR	DNA damage response
DSBs	DNA double-strand breaks
FBA	F-box associated region
FBW7	F box/WD repeat-containing protein 7
FOXN	A member of the Forkhead box transcription factors
MARCH8	Membrane-associated RING-CH
NAE	Neddylation activation enzyme
NAE1	NEDD8 activating enzyme E1
NEDD4L	Neural precursor cell expressed, developmentally down-regulated 4-like
OTUB	Ovarian tumor domain-containing ubiquitin aldehyde binding protein
Pirh2	p53-Induced ring-H2 protein
PPAR	Peroxisome proliferator-activated receptors
TSC	Tuberous sclerosis complex
UBCH	Ubiquitin-conjugating enzyme H
Ube	Ubiquitin-conjugating enzyme
UCH	Ubiquitin carboxyl-terminal hydrolase
UCP	Ubiquitin carrier protein
UHRF	Ubiquitin-like with plant homeodomain and ring finger domains
UPP	Ubiquitin proteasome pathway
UPS	Ubiquitin proteasome system
USP	Ubiquitin specific peptidase
VHL	Von Hippel-Lindau
β-Trcp	β-transducin repeat-containing protein
